# Reduced Preoperative Glomerular Filtration Rate Is Associated With Adverse Postoperative Oncological Prognosis in Patients Undergoing Radical Nephroureterectomy for Upper Tract Urothelial Carcinoma: A Retrospective Cohort Study

**DOI:** 10.3389/fsurg.2022.872273

**Published:** 2022-04-25

**Authors:** Shijie Li, Xiaonan Chen, Jianyi Zheng, Xuefeng Liu

**Affiliations:** Department of Urology, Shengjing Hospital of China Medical University, Shenyang, China

**Keywords:** upper tract urothelial carcinoma, estimated glomerular filtration rate, renal insufficiency, radical nephroureterectomy, prognostic impact

## Abstract

**Objective:**

To evaluate the association between perioperative estimated glomerular filtration rate (eGFR) and postoperative oncological outcomes in patients with upper tract urothelial carcinoma (UTUC) treated with radical nephroureterectomy (RNU),and to evaluate the effect of sex on this association.

**Methods:**

The medical records of patients with UTUC who underwent RNU between January 2012 and December 2017 at our hospital were retrospectively reviewed. Patients were divided into three groups based on preoperative eGFRs: normal eGFR (>60 mL/min/1.73 m^2^; *n* = 179), moderately reduced eGFR (45–60 mL/min/1.73 m^2^; *n* = 45), and severely reduced eGFR (≤ 45 mL/min/1.73 m^2^; *n* = 36). Statistical analyses were performed to evaluate the prognostic impact of preoperative eGFR on prognosis.

**Results:**

Patient mean age was 66.7 ± 9.6 years, and 47.9% were female. Multivariate regression analysis based on Cox proportional risk models and Kaplan-Meier survival rates showed that lower preoperative eGFR was associated with decreased OS, PFS, and CSS. In the adjusted Cox regression model, patients with normal and moderately reduced eGFRs had a decreased hazard for mortality, with adjusted hazard ratios of 0.13 [95% confidence interval (CI): 0.07–0.26] and 0.36 (95% CI: 0.18–0.73), respectively (*P* < 0.001). The smooth fitting curve suggested a linear relationship between eGFR and prognostic survival. Additionally, sensitivity subgroup analyses verified an inverse relationship between the reduced eGFR and OS. Women had a lower eGFR and worse oncological outcomes than men. A nomogram for OS was developed based on multivariate analysis with a C-index of 0.754 (95% CI: 0.728–0.779).

**Conclusion:**

Preoperative renal insufficiency is strongly associated with a higher risk of cancer progression and a lower survival probability. It is important to identify preoperative renal insufficiency in patients with UTUC, particularly female patients.

## Introduction

Upper urinary tract cancer (UTUC) is an aggressive malignancy, accounting for 5–10% of uroepithelial cancers (1, 2). Despite improved detection of early-stage tumors owing to advances in diagnostic techniques, the recurrence rate and progression rate for UTUC remain high (3). Although radical nephroureterectomy (RNU) allows longer survival, recurrence or metastasis is common after surgery, and long-term survival remains low (4). Chemotherapy and immunotherapy have been widely used in recent years to treat patients diagnosed with locally advanced or metastatic UTUC with limited efficacy (5). Early determination of patient prognosis is essential to select the optimal treatment strategy.

Currently, independent predictors of UTUC prognosis have been identified, including age (6), tumor stage (7), tumor site (8), lymphovascular infiltration (9), lymph node infiltration (10), and recurrence pattern (11). Increasing evidence indicates that preoperative renal insufficiency is correlated with adverse prognosis and malignant progression in UTUC (12, 13). Previous studies have shown a relationship between preoperative estimated glomerular filtration rate (eGFR) reduction and extra-urinary recurrence and poor oncological survival in UTUC patients (14–16). However, there is no consensus regarding the impact of preoperative renal insufficiency on tumor prognosis in patients with UTUC undergoing RNU.

The present study compared the oncological prognosis of patients with UTUC categorized according to different groups of eGFR to determine the prognostic significance of eGFR as a predictor of tumor prognosis in patients with UTUC undergoing RNU.

## Materials and Methods

### Study Cohort

**Figure 1:**
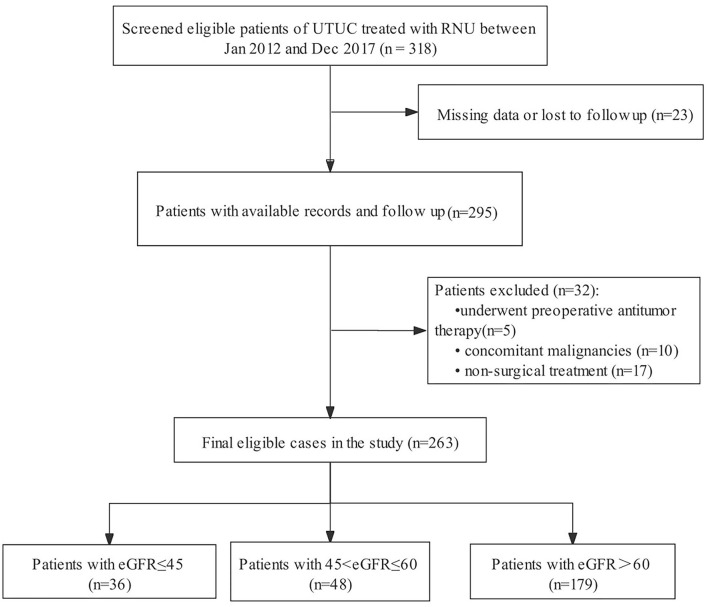
Patient selection flowchart.

We initially screened 318 consecutive admissions of patients with UTUC to our hospital between January 2012 and December 2017. The inclusion criteria were: (1) pathological confirmation of UTUC and (2) having undergone RNU. The exclusion criteria were as follows: (1) incomplete follow-up information or clinicopathological data (*n* = 23); (2) administration of preoperative anticancer therapy (*n* = 5); (3) concurrent malignancy (*n* = 10); and (4) administration of non-surgical treatment (*n* = 17). Patients were selected in strict accordance with predetermined exclusion criteria to ensure the relative homogeneity of the selected patients. Finally, 263 eligible patients were included in the analyses (Figure 1).

The study was conducted according to the Declaration of Helsinki and approved by the Ethics Committee of the Shengjing Hospital of China Medical University (ID:2021PS668K). Owing to the anonymous nature of the data, the requirement for informed consent was waived.

### Data Collection and Covariates

The following clinicopathologic information was collected from the medical records: age, sex, body mass index (BMI), comorbidities such as hypertension or coronary heart disease (CHD), tumor laterality, tumor focality, previous or concomitant bladder cancer (BC), smoking history, tumor location, tumor size, tumor stage, lymph node metastasis, tumor grade, RNU surgical approach, adjuvant chemotherapy received, and preoperative eGFR. Enhanced pelvic computed tomography (CT) was performed to determine the depth of tumor invasion. Clinical stage was determined through clinical evaluation using the American Joint Committee on Cancer TNM staging system (8th edition). Histopathological diagnosis was made according to the 1973 World Health Organization criteria.

The study population was divided into three groups according to baseline eGFR: severely reduced eGFR (≤ 45 mL/min/1.73 m^2^; *n* = 36), moderately reduced eGFR (45–60 mL/min/1.73 m^2^; *n* = 45), and normal eGFR (>60 mL/min/1.73 m^2^; *n* = 179).

### Patient Follow Up

Regular follow up was scheduled every 3–6 months in the first 5 postoperative years and yearly thereafter. Local recurrence and distant metastasis were diagnosed using imaging and pathology. The primary end point was overall survival (OS); progression-free survival (PFS) and cancer-specific survival (CSS) were secondary end points. Patients were followed up until death or the cutoff date of December 2017.

### Statistical Analyses

Continuous variables are expressed as mean (SD) for normally distributed variables and as median (interquartile range) for non-normally distributed variables. The Mann-Whitney U test and chi-square test or Fisher's exact test were used to test the correlation between the groups of variables. Survival curves were plotted using the Kaplan-Meier method and compared using a log-rank test. Multivariable analysis using the Cox proportional hazards model assessed the influence of eGFR on PFS, CSS, and OS.

Our analysis was adjusted and stratified for all potential confounders to explore the association between eGFR and tumor prognosis (linear or non-linear) and to determine the factors influencing this correlation. Cox proportional-hazards models were developed to examine the association of eGFR detected with PFS, CSS, and OS and used for multivariate analysis. The models in this study used three predefined models of adjustment (model 1: unadjusted; model 2: adjusted for age, sex, BMI; and model 3: adjusted for age, sex, BMI, hypertension, CHD, diabetes, smoking history, history of BC, concomitant BC, tumor laterality, tumor location, tumor focality, tumor size; and model 4: a final model adjusted for all covariates simultaneously). A penalized regression spline approach was used to investigate whether there was a potential non-linear relationship between eGFR and tumor survival. The hazard ratios (HRs) were estimated using a stratified Cox proportional hazards model, and the likelihood ratio test was used to detect the presence of subgroup interactions. Adjustment models and curve fitting were similarly used to explore whether there were differences in eGFR and survival outcomes according to sex. Furthermore, based on multivariate Cox regression models, a nomogram was constructed for 3- and 5-year OS. The C-index of the nomogram was calculated to show the discriminative ability. Calibration curves were created to compare the predicted probabilities of the nomogram with the observed results.

The statistical software package R v.4.0.2 (http://www.R-project.org, The R Foundation) and Free Statistics software version 1.4 were used for statistical analyses. Statistical significance was set at a two-tailed *P* < 0.05.

## Results

### Baseline Clinicopathologic Characteristics

Table 1 showed the demographic and clinicopathological features of 263 patients with UTUC in the groups based on eGFR, including 137 (52.1%) men and 126 (47.9%) women. There was a higher proportion of patients with severely reduced eGFR among female patients (*P* = 0.008). The number of patients with CHD, positive lymph node, and anemia was higher in the severely reduced eGFR group compared with those in the normal and moderately reduced eGFR group (*P* < 0.05). There were no significant differences in age, BMI, hypertension, diabetes, smoking history, tumor laterality, previous or concomitant BC, tumor location, tumor size, tumor grade, T stage, surgical approach, adjuvant chemotherapy received, and hypoalbuminemia among the different eGFR groups (all *P* > 0.05).

**Table 1:** The relationship between eGFR groups and clinicopathological parameters in the UTUC cohort (*n* = 263).

**Characteristics**	**Total**	**eGFR ≤45**	**45 < eGFR ≤60**	**eGFR >60**	***P*-value**
Number of patients	263	36	48	179	
Age (years), Mean ± SD	66.7 ± 9.6	70.1 ± 9.5	66.2 ± 9.3	66.1 ± 9.6	0.065
Sex, *n* (%)					0.008^*^
Male	137 (52.1)	11 (30.6)	22 (45.8)	103 (57.5)	
Female	126 (47.9)	25 (69.4)	26 (54.2)	76 (42.5)	
BMI, Mean ± SD	23.8 ± 3.8	23.8 ± 4.2	24.1 ± 3.4	23.7 ± 3.9	0.789
Hypertension, *n* (%)					0.217
No	171 (65.0)	28 (77.8)	31 (64.6)	112 (62.6)	
Yes	92 (35.0)	8 (22.2)	17 (35.4)	67 (37.4)	
CHD, *n* (%)					
No	218 (82.9)	27 (75)	35 (72.9)	156 (87.2)	0.027^*^
Yes	45 (17.1)	9 (25)	13 (27.1)	23 (12.8)	
Diabetes, *n* (%)					0.254
No	231 (87.8)	34 (94.4)	44 (91.7)	153 (85.5)	
Yes	32 (12.2)	2 (5.6)	4 (8.3)	26 (14.5)	
Smoking history, *n* (%)					0.395
No	180 (68.4)	22 (61.1)	36 (75)	122 (68.2)	
Yes	83 (31.6)	14 (38.9)	12 (25)	57 (31.8)	
History of BC, *n* (%)					0.723
No	256 (97.3)	35 (97.2)	46 (95.8)	175 (97.8)	
Yes	7 (2.7)	1 (2.8)	2 (4.2)	4 (2.2)	
Concomitant BC, *n* (%)					0.917
No	249 (94.7)	34 (94.4)	45 (93.8)	170 (95)	
Yes	14 (5.3)	2 (5.6)	3 (6.2)	9 (5)	
Laterality, *n* (%)					0.224
Left	144 (54.8)	24 (66.7)	23 (47.9)	97 (54.2)	
Right	119 (45.2)	12 (33.3)	25 (52.1)	82 (45.8)	
Location, *n* (%)					0.096
Renal pelvis	105 (39.9)	10 (27.8)	14 (29.2)	81 (45.3)	
Ureter	140 (53.2)	23 (63.9)	29 (60.4)	88 (49.2)	
Multiple	18 (6.8)	3 (8.3)	5 (10.4)	10 (5.6)	
Size (cm), Median (IQR)	2.6 (1.6, 3.6)	2.2 (1.6, 3.0)	2.4 (1.5, 3.5)	2.9 (1.8, 4.0)	0.313
Tumor grade, *n* (%)					0.099
Low	55 (20.9)	3 (8.3)	9 (18.8)	43 (24)	
High	208 (79.1)	33 (91.7)	39 (81.2)	136 (76)	
T stage, *n* (%)					0.106
T1	93 (35.4)	11 (30.6)	18 (37.5)	64 (35.8)	
T2	129 (49.0)	20 (55.6)	17 (35.4)	92 (51.4)	
T3	41 (15.6)	5 (13.9)	13 (27.1)	23 (12.8)	
Lymph node status, *n* (%)					0.002^*^
Negative	246 (93.5)	29 (80.6)	44 (91.7)	173 (96.6)	
Positive	17 (6.5)	7 (19.4)	4 (8.3)	6 (3.4)	
Surgical approach, *n* (%)					0.042^*^
Laparoscopic	96 (36.5)	13 (36.1)	25 (52.1)	58 (32.4)	
Open	167 (63.5)	23 (63.9)	23 (47.9)	121 (67.6)	
Chemotherapy, *n* (%)					0.604
No	176 (66.9)	23 (63.9)	35 (72.9)	118 (65.9)	
Yes	87 (33.1)	13 (36.1)	13 (27.1)	61 (34.1)	
Hypoalbuminemia, *n* (%)					0.508
No	224 (85.2)	29 (80.6)	43 (89.6)	152 (84.9)	
Yes	39 (14.8)	7 (19.4)	5 (10.4)	27 (15.1)	
Anemia, *n* (%)					0.004^*^
No	203 (77.2)	20 (55.6)	39 (81.2)	144 (80.4)	
Yes	60 (22.8)	16 (44.4)	9 (18.8)	35 (19.6)	
eGFR (mL/min/1.73 m^2^), Mean ± SD	75.0 ± 28.2	34.6 ± 7.4	52.8 ± 4.4	89.0 ± 22.2	<0.001^*^

* *P < 0.05. eGFR, estimated glomerular filtration rate; BMI, body mass index; CHD, coronary heart disease; UTUC, upper tract urothelial carcinoma; BC, bladder cancer*.

The median follow-up period was 36 months [interquartile range (IQR): 1–91 months]. At the time of the last follow up, 60 (22.8%) patients died of UTUC, postoperative tumor progression was confirmed in 73 (27.8%) patients, and 61 (25.5%) patients died due to all causes. The estimated 5-year CSS, OS, and PFS were 71.5, 68.1, and 66.0%, respectively.

### Univariate and Multivariate Cox Regression Analysis

**Figure 2:**
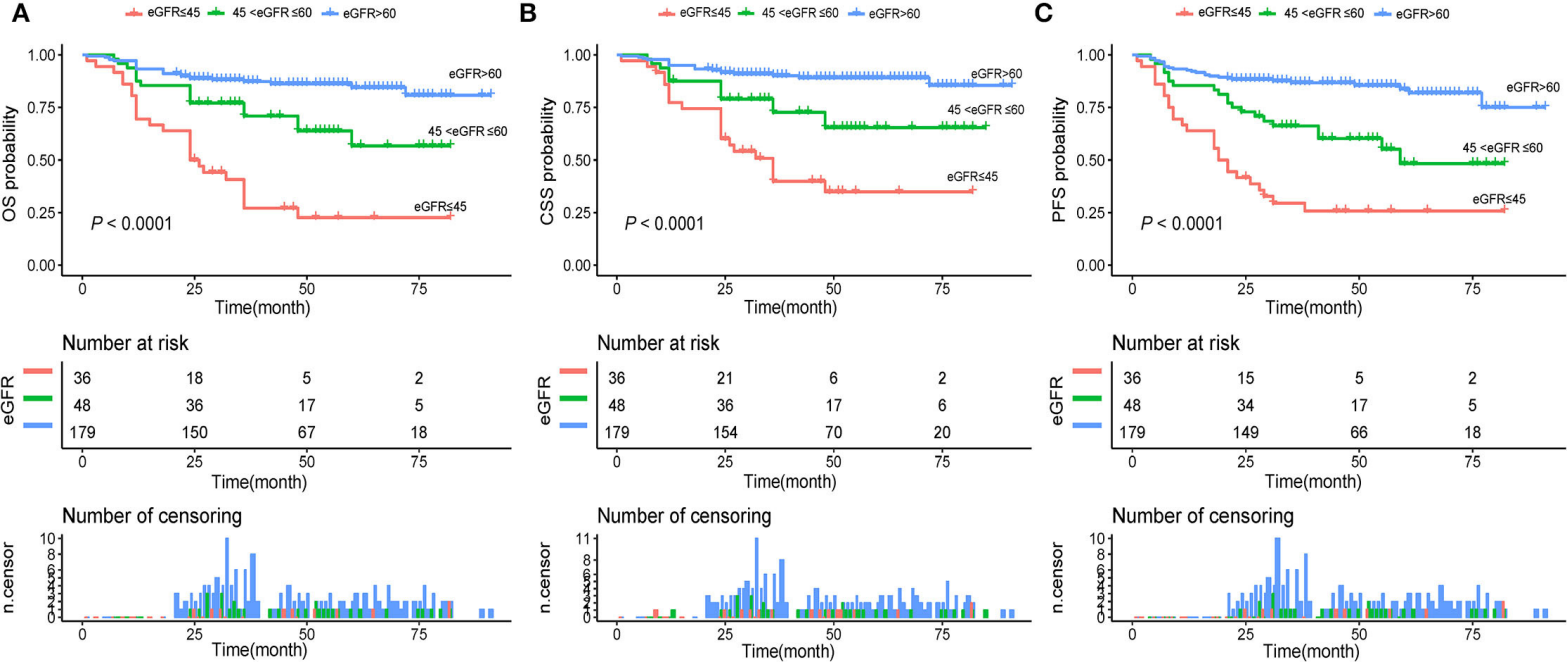
**(A)** Kaplan–Meier curves for OS of patients with UTUC stratified by postoperative eGFR groups; **(B)** Kaplan–Meier curves for CSS of patients with UTUC stratified by postoperative eGFR groups; **(C)** Kaplan–Meier curves for PFS of patients with UTUC stratified by postoperative eGFR groups. UTUC, upper tract urothelial carcinoma; eGFR, estimated glomerular filtration rate; OS, overall survival; CSS, cancer-specific survival; PFS, progression free survival.

Univariable and multivariable Cox regression were used to analyze the association between selected clinicopathological features and oncological survival. After univariate analysis, the statistically significant factors were included in the multivariate analysis. Finally, the multivariate analysis indicated that sex, CHD, smoking history, and moderately and severely reduced eGFR were significantly associated with unfavorable CSS (*P* < 0.05, Table 2). Sex, BMI, CHD, smoking history, and moderately and severely reduced eGFR were correlated with unfavorable OS and PFS (all *P* < 0.05). In addition, Kaplan-Meier curves showed a significant association between the severely and moderately reduced eGFR group and worse OS, CSS, and PFS (Figures 2A–C).

**Table 2:** Univariate and multivariate Cox regression analyses of clinicopathological parameters for the prediction of survival outcomes in patients with UTUC treated with RNU.

**Covariates**	**OS**				**CSS**				**PFS**			
	**Univariate**		**Multivariate**		**Univariate**		**Multivariate**		**Univariate**		**Multivariate**	
	**HR (95% CI)**	***P*** **-value**	**HR (95% CI)**	***P*** **-value**	**HR (95% CI)**	***P*** **-value**	**HR (95% CI)**	***P*** **-value**	**HR (95% CI)**	***P*** **-value**	**HR (95% CI)**	***P*** **-value**
Age (≥67 vs. <67 years)	1.31 (0.81, 2.13)	0.271			1.30 (0.78, 2.17)	0.309			1.28 (0.81, 2.04)	0.293		
Sex (female vs. male)	1.81 (1.10, 2.95)	0.018^*^	1.82 (1.09, 3.03)	0.022^*^	1.98 (1.17, 3.34)	0.011^*^	1.98 (1.14, 3.44)	0.016^*^	1.74 (1.09, 2.79)	0.020^*^	1.73 (1.06~2.81)	0.027^*^
BMI (≥23.95 vs. <23.95)	1.76 (1.08, 2.88)	0.024^*^	1.76 (1.06, 2.90)	0.028^*^	1.67 (0.99, 2.80)	0.053^*^	1.05 (0.98, 1.11)	0.159	1.79 (1.11, 2.87)	0.016^*^	1.78 (1.1~2.87)	0.019^*^
Hypertension (yes vs. no)	0.86 (0.51, 1.44)	0.561			0.80 (0.46, 1.40)	0.439			0.94 (0.57, 1.53)	0.794		
CHD (yes vs. no)	2.15 (1.26, 3.65)	0.005^*^	1.89 (1.10, 3.26)	0.022^*^	2.31 (1.33, 4.02)	0.003^*^	1.97 (1.11, 3.48)	0.020^*^	2.08 (1.24, 3.47)	0.005^*^	1.76 (1.04~2.98)	0.036^*^
Diabetes (yes vs. no)	1.03 (0.49, 2.16)	0.938			1.01 (0.46, 2.21)	0.99			0.95 (0.46, 1.99)	0.897		
Smoking (yes vs. no)	2.04 (1.26, 3.29)	0.004^*^	2.18 (1.30, 3.63)	0.003^*^	2.07 (1.25, 3.44)	0.005^*^	2.28 (1.32, 3.94)	0.003^*^	1.85 (1.17, 2.94)	0.009^*^	2.03 (1.24~3.31)	0.005^*^
History of BC (yes vs. no)	0.45 (0.06, 3.26)	0.431			0 (0, Inf)	0.996			0.42 (0.06, 3.01)	0.386		
Concomitant BC (yes vs. no)	1.43 (0.57, 3.55)	0.445			0.92 (0.29, 2.95)	0.893			1.36 (0.55, 3.38)	0.505		
Laterality (right vs. left)	0.82 (0.50, 1.33)	0.425			0.77 (0.46, 1.28)	0.31			0.84 (0.53, 1.34)	0.475		
Tumor location (ureteric vs. pelvicalyceal)	1.22 (0.73, 2.03)	0.457			1.31 (0.76, 2.25)	0.325			1.24 (0.76, 2.03)	0.389		
Tumor location (multiple vs. pelvicalyceal)	1.44 (0.59, 3.53)	0.425			1.10 (0.38, 3.20)	0.864			1.37 (0.56, 3.34)	0.485		
Tumor size (≥2.6 cm vs. <2.6 cm)	0.99 (0.61, 1.60)	0.972			0.91 (0.55, 1.51)	0.709			0.95 (0.6, 1.5)	0.813		
Tumor grade (high vs. low)	2.80 (1.21, 6.48)	0.016^*^	1.92 (0.82, 4.54)	0.135	3.03 (1.21, 7.56)	0.018^*^	1.94 (0.76, 4.96)	0.168	2.33 (1.12, 4.86)	0.024^*^	1.64 (0.77~3.49)	0.196
pT2 vs. pT1	1.10 (0.64, 1.91)	0.722			1.01 (0.57, 1.79)	0.962			1.08 (0.64, 1.83)	0.776		
pT3 vs. pT1	1.44 (0.72, 2.88)	0.299			1.27 (0.61, 2.65)	0.523			1.55 (0.81, 2.97)	0.189		
Lymph node status (positive vs. negative)	2.68 (1.33, 5.42)	0.006^*^	1.14 (0.54, 2.41)	0.728	3.06 (1.50, 6.22)	0.002^*^	1.28 (0.60, 2.71)	0.523	2.55 (1.26, 5.15)	0.009^*^	1.14 (0.54~2.39)	0.728
Surgery (open vs. laparoscopic)	1.09 (0.66, 1.81)	0.731			0.92 (0.55, 1.55)	0.751			1.05 (0.65, 1.7)	0.837		
Chemotherapy (yes vs. no)	1.22 (0.74, 2.00)	0.443			1.26 (0.75, 2.14)	0.381			1.26 (0.78, 2.03)	0.34		
Hypoalbuminemia (yes vs. no)	1.02 (0.52, 2.00)	0.952			0.90 (0.43, 1.89)	0.771			0.93 (0.48, 1.82)	0.843		
Anemia (yes vs. no)	1.45 (0.85, 2.47)	0.168			1.45 (0.83, 2.54)	0.197			1.51 (0.91, 2.51)	0.112		
eGFR(45 < eGFR ≤ 60 vs. ≤ 45)	0.34 (0.18, 0.63)	<0.001^*^	0.44 (0.23, 0.84)	0.013^*^	0.32 (0.17, 0.60)	<0.001^*^	0.43 (0.22, 0.85)	0.014^*^	0.41 (0.23, 0.74)	0.003^*^	0.52 (0.28~0.95)	0.035^*^
eGFR (eGFR>60 vs. ≤ 45)	0.13 (0.07, 0.22)	<0.001^*^	0.16 (0.09, 0.29)	<0.001^*^	0.10 (0.06, 0.18)	<0.001^*^	0.14 (0.07, 0.26)	<0.001^*^	0.14 (0.08, 0.23)	<0.001^*^	0.18 (0.1~0.32)	<0.001^*^

* *P < 0.05. OS, overall survival; CSS, cancer-specific survival; PFS, progression free survival; HR, hazard ratio; CI, confidence interval; eGFR, estimated glomerular filtration rate; BMI, body mass index; CHD, coronary heart disease; UTUC, upper tract urothelial carcinoma; BC, bladder cancer*.

### Adjusted Cox Proportional Hazards Analyses for eGFR

The independent effect of eGFR on OS was determined by constructing four models (Table 3). In the unadjusted model, the increased risk of death was negatively associated with the continuous eGFR or the eGFR group. In model 1 adjusted for age, sex, and BMI, patients in both the moderately reduced and normal eGFR groups had a significantly lower risk of death compared with those in the severely reduced eGFR group [HR = 0.36, 95% confidence interval (CI): 0.19–0.67; HR = 0.15, 95% CI: 0.08–0.26]; this trend persisted in the fully adjusted model 3 (HR = 0.43, 95% CI: 0.22–0.87 and HR = 0.17, 95% CI: 0.09–0.32).

**Table 3:** Multiple Cox regrssion analysis of eGFR in patients with UTUC.

**eGFR**	**Non-adjusted**	***P*-value**	**Adjust I**	***P*-value**	**Adjust II**	***P*-value**	**Adjust III**	***P*-value**
Continuous	0.97 (0.96, 0.98)	<0.0001^*^	0.97 (0.96, 0.98)	<0.0001^*^	0.97 (0.96, 0.98)	<0.0001^*^	0.97 (0.96, 0.98)	<0.0001^*^
Group								
eGFR ≤ 45	1 (reference)		1 (reference)		1 (reference)		1 (reference)	
45 < eGFR ≤ 60	0.35 (0.19, 0.65)	0.0010^*^	0.36 (0.19, 0.67)	0.0015^*^	0.43 (0.22, 0.83)	0.0120^*^	0.43 (0.22, 0.87)	0.0187^*^
eGFR >60	0.14 (0.08, 0.24)	<0.0001^*^	0.15 (0.08, 0.26)	<0.0001^*^	0.15 (0.08, 0.28)	<0.0001^*^	0.17 (0.09, 0.32)	<0.0001^*^

*Non-adjusted model adjusted for: none*.

*Adjust I model adjusted for: age, sex, BMI*.

*Adjust II model adjusted for: age, sex, BMI, hypertension, CHD, diabetes, smoking history, history of BC, concomitant of BC, tumor laterality, tumor location, tumor focality, tumor size*.

*Adjust III model adjusted for: age, sex, BMI, hypertension, CHD, diabetes, smoking history, history of BC, concomitant of BC, tumor laterality, tumor location, tumor focality, tumor size, tumor grade, pT stage, pN stage, surgical approach, chemotherapy, hypoalbuminemia, anemia*.

* *P < 0.05. BMI, body mass index; eGFR, estimated glomerular filtration rate; BC, bladder cancer; CHD; coronary heart disease; UTUC, upper tract urothelial carcinoma*.

### Time-Dependent ROC Analysis of eGFR for 3- and 5-Year OS, CSS, and PFS

**Figure 3:**
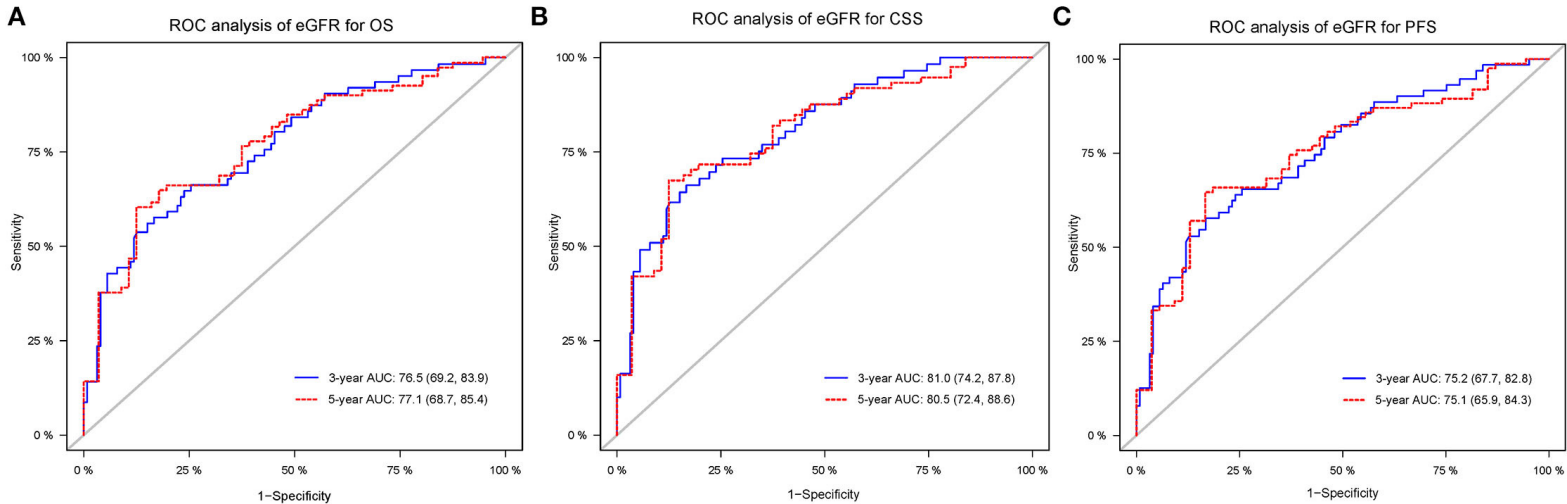
Receiver operating characteristic curves of postoperative eGFR for predicting OS **(A)**, CSS **(B)**, PFS **(C)** in the primary cohort. eGFR, estimated glomerular filtration rate; OS, overall survival; CSS, cancer-specific survival; PFS, progression free survival.

As shown in Figure 3A, time-dependent ROC analysis showed that the 3- and 5-year AUC for OS was 0.765 (95 % CI: 0.692–0.839) and 0.771 (95 % CI: 0.687–0.854), respectively, indicating a promising value of eGFR for predicting OS. Similarly, time-dependent ROC analysis of eGFR also indicated a superior ability in predicting CSS and PFS in patients with UTUC (Figures 3B,C).

### Exploration of the Non-linear Relationship Between eGFR and Survival

**Figure 4:**
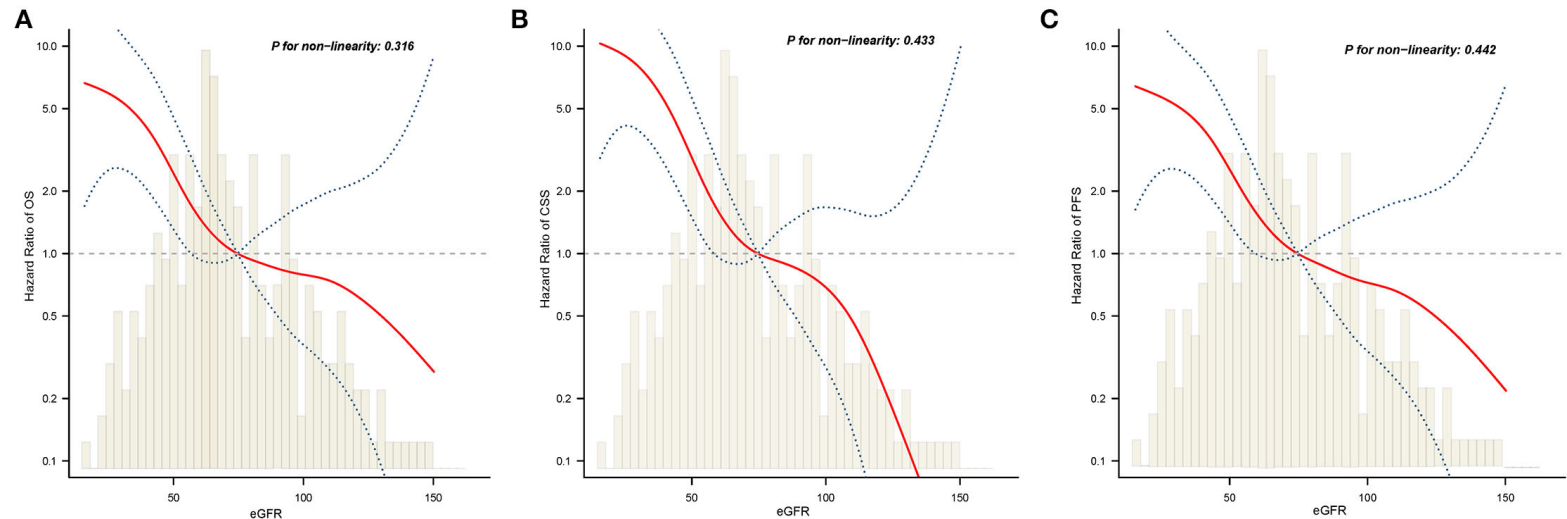
The adjusted smooth fitting curve between postoperative eGFR and postoperative survival outcomes of UTUC patients. A linear relationship between postoperative eGFR and OS **(A)**, CSS **(B)**, PFS **(C)** was observed (all *P* > 0.05). The red solid line and blue dashed line represent the estimated values and their corresponding 95% confidence intervals. UTUC, upper tract urothelial carcinoma; eGFR, estimated glomerular filtration rate; OS, overall survival; CSS, cancer-specific survival; PFS, progression free survival.

Next, we examined whether there was a non-linear correlation between low eGFR and worse OS, CSS, or PFS (Figures 4A–C). After adjustment for potential confounders, the smooth curves showed negative linear correlation between eGFR and oncological outcomes. Results from two piecewise linear regression and recursive algorithms showed a relationship between eGFR and risk of outcome, with no saturation or threshold effects (likelihood ratio test *P* > 0.05).

### Subgroup Analyses

Subgroup analysis was performed by stratifying all covariates to further confirm that the study results were reliable in the presence of underlying confounding factors. Age, sex, BMI, comorbidities such as hypertension or CHD, tumor laterality, previous or concomitant BC, smoking history, tumor size, tumor location, tumor grade, pT stage, pN stage, RNU surgical approach, and adjuvant chemotherapy were stratified (Supplementary Table 1). Compared with the severely reduced eGFR group, the moderately reduced and normal eGFR group showed a decreasing trend of HRs among all the subgroups.

### Effect of Sex on the Prognosis of UTUC

**Figure 5:**
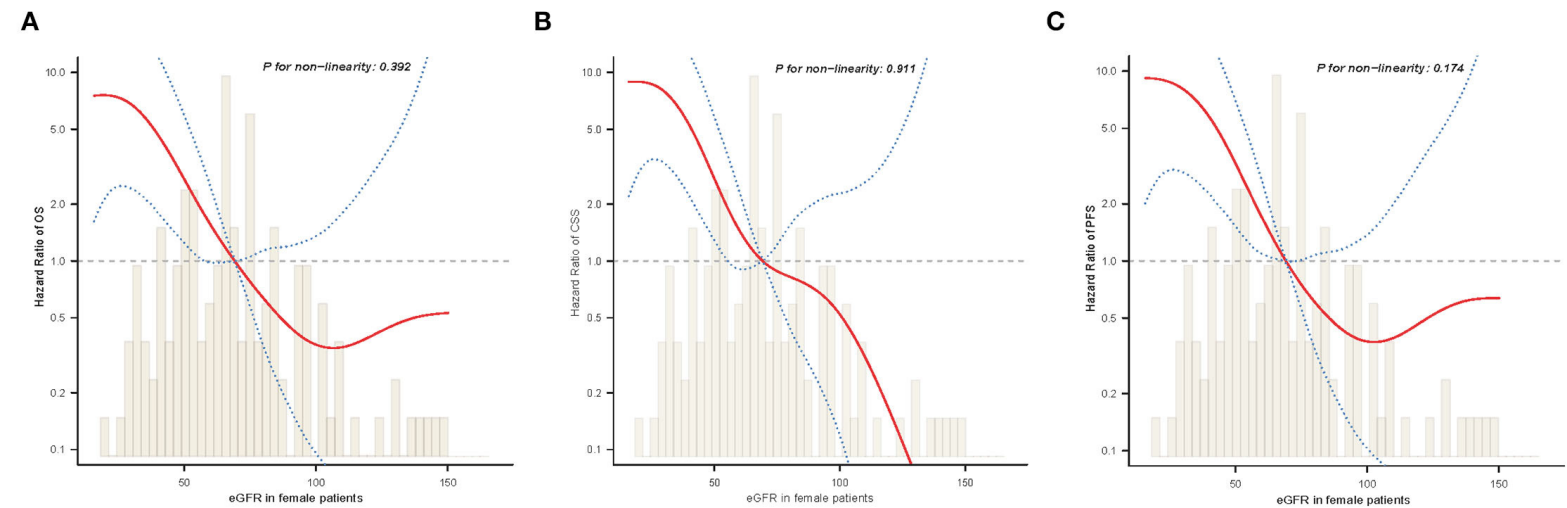
The adjusted smooth fitting curve between postoperative eGFR and postoperative survival outcomes of female UTUC patients. A linear relationship between postoperative eGFR and OS **(A)**, CSS **(B)**, PFS **(C)** was observed (all *P* > 0.05). The red solid line and blue dashed line represent the estimated values and their corresponding 95% confidence intervals. UTUC, upper tract urothelial carcinoma; eGFR, estimated glomerular filtration rate; OS, overall survival; CSS, cancer-specific survival; PFS, progression free survival.

As shown in Table 2, significant differences were observed between male and female estimates in postoperative oncological prognosis as assessed by multivariate Cox regression models for OS (HR = 1.82, 95% CI: 1.09–3.03, *P* = 0.022), CSS (HR = 1.98, 95% CI: 1.14–3.44, *P* = 0.016), and PFS (HR = 1.73, 95% CI: 1.06–2.81, *P* = 0.027). The association of sex with clinicopathologic features was further analyzed. As shown in Table 4, the median age of female and male patients was 68.3 ± 9.7 and 65.2 ± 9.2 years, respectively (*P* = 0.009). Significant sex-related differences were also found for CHD (*P* = 0.011), smoking history (*P* < 0.001), T stage (*P* = 0.030), anemia (*P* = 0.506), and eGFR (*P* = 0.001). In addition, we analyzed whether there was a non-linear relationship between low eGFR and poor OS, CSS, or PFS in female patients (Figures 5A–C). After adjusting for possible confounders, the smoothed curves showed a linear correlation between low eGFR and poor oncological prognosis in female patients with UTUC (likelihood ratio test *P* > 0.05).

**Table 4:** Association of sex with clinical and pathological characteristics in UTUC patients treated with RNU (*n* = 263).

**Characteristics**	**Total**	**Male (*n* = 136)**	**Female (*n* = 127)**	***P*-value**
Age (years), Mean ± SD	66.7 ± 9.6	65.2 ± 9.2	68.3 ± 9.7	0.009^*^
BMI, Mean ± SD	23.8 ± 3.8	23.8 ± 3.6	23.8 ± 4.1	0.996
Hypertension, *n* (%)				0.426
No	171 (65.0)	92 (67.6)	79 (62.2)	
Yes	92 (35.0)	44 (32.4)	48 (37.8)	
CHD, *n* (%)				
No	218 (82.9)	121 (89)	97 (76.4)	0.011^*^
Yes	45 (17.1)	15 (11)	30 (23.6)	
Diabetes, *n* (%)				0.986
No	231 (87.8)	120 (88.2)	111 (87.4)	
Yes	32 (12.2)	16 (11.8)	16 (12.6)	
Smoking history, *n* (%)				<0.001^*^
No	180 (68.4)	80 (58.8)	100 (78.7)	
Yes	83 (31.6)	56 (41.2)	27 (21.3)	
History of BC, *n* (%)				0.268
No	256 (97.3)	134 (98.5)	122 (96.1)	
Yes	7 (2.7)	2 (1.5)	5 (3.9)	
Concomitant BC, *n* (%)				0.132
No	249 (94.7)	132 (97.1)	117 (92.1)	
Yes	14 (5.3)	4 (2.9)	10 (7.9)	
Laterality, *n* (%)				0.317
Left	144 (54.8)	79 (58.1)	65 (51.2)	
Right	119 (45.2)	57 (41.9)	62 (48.8)	
Location, *n* (%)				0.414
Renal pelvis	105 (39.9)	58 (42.6)	47 (37)	
Ureter	140 (53.2)	71 (52.2)	69 (54.3)	
Multiple	18 (6.8)	7 (5.1)	11 (8.7)	
Size (cm), Median (IQR)	2.6 (1.6, 3.6)	2.5 (1.5, 3.5)	3.0 (2.0, 4.0)	0.111
Tumor grade, *n* (%)				0.218
Low	55 (20.9)	33 (24.3)	22 (17.3)	
High	208 (79.1)	103 (75.7)	105 (82.7)	
T stage, *n* (%)				0.030^*^
T1	93 (35.4)	56 (41.2)	37 (29.1)	
T2	129 (49.0)	56 (41.2)	73 (57.5)	
T3	41 (15.6)	24 (17.6)	17 (13.4)	
Lymph node status, *n* (%)				0.517
Negative	246 (93.5)	129 (94.9)	117 (92.1)	
Positive	17 (6.5)	7 (5.1)	10 (7.9)	
Surgical approach, *n* (%)				0.079
Laparoscopic	96 (36.5)	57 (41.9)	39 (30.7)	
Open	167 (63.5)	79 (58.1)	88 (69.3)	
Chemotherapy, *n* (%)				0.696
No	176 (66.9)	93 (68.4)	83 (65.4)	
Yes	87 (33.1)	43 (31.6)	44 (34.6)	
Hypoalbuminemia, *n* (%)				0.418
No	224 (85.2)	113 (83.1)	111 (87.4)	
Yes	39 (14.8)	23 (16.9)	16 (12.6)	
Anemia, *n* (%)				0.002^*^
No	203 (77.2)	116 (85.3)	87 (68.5)	
Yes	60 (22.8)	20 (14.7)	40 (31.5)	
eGFR (mL/min/1.73 m^2^), Median (IQR)	70.5 (54.0, 94.9)	76.0 (61.0, 97.9)	66.2 (49.1, 83.5)	0.001^*^

* *P < 0.05. eGFR, estimated glomerular filtration rate; BMI, body mass index; CHD, coronary heart disease; UTUC, upper tract urothelial carcinoma; BC, bladder cancer*.

In addition, we further determined the independent effects of gender on OS, CSS, and PFS by constructing three models. As shown in Table 5, postoperative oncological outcomes were significantly worse in women in unadjusted as well as adjusted models (all *P* < 0.05).

**Table 5:** Survival after radical surgery: multivariable analysis comparing female with male patients.

**End point**	**Events (%)**	**Crude**	**Adjust I**		**Adjust II**		
		**HR (95% CI)**	***P*** **-value**	**HR (95% CI)**	***P*** **-value**	**HR (95% CI)**	***P*** **-value**
OS							
Male	26 (19.1)	1 (reference)		1 (reference)		1 (reference)	
Female	41 (32.3)	1.79 (1.10, 2.93)	0.0198^*^	2.00 (1.18, 3.41)	0.0101^*^	2.28 (1.28, 4.04)	0.0050^*^
CSS							
Male	22 (16.2)	1 (reference)		1 (reference)		1 (reference)	
Female	38 (29.9)	1.64 (0.95, 2.82)	0.0752^*^	1.90 (1.06, 3.40)	0.0312^*^	2.09 (1.13, 3.90)	0.0197^*^
PFS							
Male	29 (21.3)	1 (reference)		1 (reference)		1 (reference)	
Female	44 (34.6)	1.74 (1.09, 2.78)	0.0209^*^	1.91 (1.15, 3.17)	0.0118^*^	2.11 (1.22, 3.63)	0.0072^*^

* *P < 0.05*.

*Crude model adjusted for: none*.

*Adjust I model adjusted for: age, body mass index, hypertension, coronary heart disease, diabetes, smoking history, history of bladder cancer, concomitant of BC, tumor laterality, tumor location, tumor focality, tumor size*.

*Adjust II model adjusted for all confounders: age, body mass index, hypertension, coronary heart disease, diabetes, smoking history, history of bladder cancer, concomitant of bladder cancer, tumor laterality, tumor location, tumor focality, tumor size, tumor grade, pT stage, pN stage, surgical approach, chemotherapy, hypoalbuminemia, anemia*.

*OS, overall survival; CSS, cancer-specific survival; PFS, progression free survival; HR, hazard ratio; CI, confidence interval*.

### Construction of Prognostic Nomogram for OS Based on eGFR

**Figure 6:**
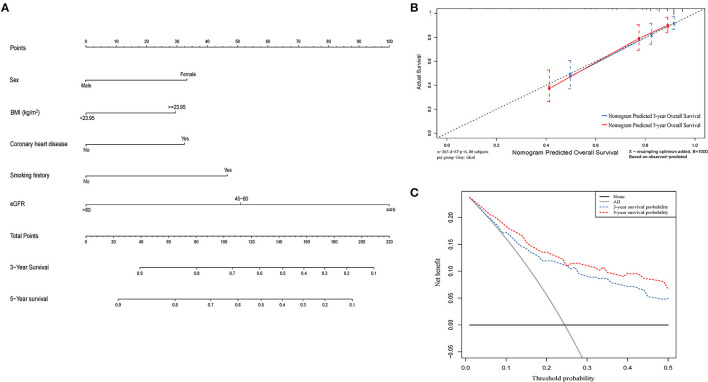
A nomogram model constructed by independent prognostic factors predicting 3- and 5-year OS for UTUC patients **(A)**; The calibration curves of the nomogram for 3- and 5- year OS **(B)**; Decision curve analysis of the nomogram for 3- and 5-year OS **(C)**. OS, overall survival; UTUC, upper tract urothelial carcinoma.

Independent risk factors identified through multivariate Cox regression analysis were used to construct a nomogram to predict OS at 3 and 5 years (Figure 6A). The C-index of the eGFR-based nomogram was 0.754 (95% CI: 0.728–0.779). Calibration plots indicated a stable consistency between the probabilities predicted by the nomogram and the actual observed values of 3- and 5-year OS in the cohort (Figure 6B). Decision curve analysis (DCA) also showed a significant net benefit at most threshold probabilities and improved performance in predicting 3- and 5-year OS (Figure 6C).

## Discussion

The main purpose of this study was to investigate the prognostic impact of preoperative renal insufficiency on prognosis in patients with UTUC treated with RNU. Our findings demonstrated that renal insufficiency was associated with poor OS, CSS, and PFS in the multivariate risk model. Moderately and severely reduced eGFRs were defined as independent risk factors for postoperative survival. This relationship remained unchanged after adjustment for potential confounding variables. In addition, our study indicated that women have a worse postsurgical oncological prognosis.

The impact of preoperative status on postoperative surgical outcomes in patients undergoing RNU remains controversial. Chronic kidney disease, hypertensive disease, diabetes mellitus, and vascular disease are commonly known risk factors that negatively affect postoperative outcomes (17–20). Previous studies have established a relationship between renal insufficiency and cancer risks; however, the association between the severity of renal insufficiency and the malignant potential of UTUC remains unclear. Few studies have evaluated the impact of preoperative severe renal insufficiency (eGFR <45 mL/min/1.73 m^2^) on survival outcomes in patients with UTUC. In a retrospective population-based cohort study of more than 1 million individuals between 2000 and 2008, the authors reported a 48% increased risk of uroepithelial carcinoma in patients with eGFR <30 mL/min/1.73 m^2^ compared with the eGFR of 60–89 mL/min/1.73 m^2^ (21). Even in patients with mildly impaired renal function, changes in eGFR can lead to the development of more aggressive cancers, resulting in high rates of recurrence and mortality (15, 22, 23). The results of this study highlight the importance of severe renal insufficiency in oncological outcomes, revealing that OS, CSS, and PFS exhibit a decrease in preoperative eGFR <60 mL/min/1.73 m^2^ and a significant deterioration in survival at eGFR ≤ 45 mL/min/1.73 m^2^.

The adverse effects of preoperative renal insufficiency on the postoperative period in patients with UTUC may be affected by a combination of many factors, including age, chronic inflammation, oxidative stress, and metabolic disturbances (24–26). In addition, decreased renal function makes the selection or therapeutic dose of agents difficult (27). Defective immune responses have been reported to be common among patients with chronic kidney disease. The functional role of monocytes and macrophages is diminished in these patients despite elevated cytokine levels (27).

In our study, we noted that pathological stage and lymph node metastases have failed to show an insignificant association with clinical outcome. Referring to the previous literature, it was controversial that whether pathological stage and lymph node metastasis could be used as independent prognostic factors of UTUC. Several studies still concluded that pathological stage or lymph node status was not related to UTUC prognosis by multivariate analysis (28–32). The reasons for these findings vary, including sample size, rate of advanced patients, and follow-up time. The underlying mechanism between pathological stage and lymph node metastasis and UTUC needs to be further studied.

Interestingly, our results also suggested a sex-specific difference in patients with UTUC treated with RNU. There is no global consensus on the impact of sex on clinicopathological features and tumor prognosis in UTUC, given the conflicting results reported earlier (33–35). Studies have shown that women are more likely to have already reached the advanced stage at the time of diagnosis and have higher cause-specific and all-cause mortality than men (34). There is limited understanding of the mechanisms underlying the finding of a worse prognosis amongst female patients with UTUC, including the potential differences in environmental exposure, genetic differences, anatomical or physiological differences, and inequalities in health care (35). Our data were consistent with previous studies showing that female sex was an independent risk factor for poor prognosis. In addition, this study revealed a significant difference between the sexes in the distribution of eGFR, with women having a lower eGFR than men (*P* = 0.008). This finding suggested that lower preoperative eGFR was relevant to poor oncologic prognosis among female patients with UTUC. Therefore, valuing and improving the preoperative eGFR in patients with UTUC, particularly female patients, may have a beneficial effect on their survival.

Several studies have constructed predictive nomograms for UTUC prognosis based on clinical and pathological variables (36–39). However, preoperative eGFR has not been included in the risk assessment models. Therefore, we sought to develop a prognostic model based on eGFR in order to develop a specific prognostic nomogram for patients presenting with preoperative renal impairment. The calibration curve and DCA analysis showed that the nomogram accurately predicted OS in patients with UTUC undergoing radical surgery, with a C-index of 0.754 (95% CI: 0.728–0.779).

Nevertheless, this study has several limitations. First, although our analysis included several variables, this study was limited by the small number of patients in the examined cohort and the retrospective design. Second, assessments regarding renal function are simple and inexpensive, but these parameters can be influenced by biochemical processes in various metabolic ways. In addition, the effect of race on eGFR may be inconsistent and may impact the representativeness of these results. However, in terms of finding the ideal method, eGFR seems to provide the best results for the measurement of renal insufficiency. Further prospective studies should be conducted to elucidate the underlying mechanisms of carcinogenesis and renal insufficiency, determine the prognostic usefulness of renal insufficiency in specific cancer types, and subsequently assess the potential for targeted therapies.

## Conclusion

Preoperative eGFR is a simple potential predictive tool for oncologic prognosis after RNU in patients with UTUC and can be used as a supplement to the surgeon's clinical judgment and experience. Sex is an independent prognostic factor affecting RNU for UTUC. Women tend to have lower eGFR and worse OS, CSS, and PFS than men. Further studies are needed to assess the impact of renal insufficiency on the prognosis of UTUC.

## Data Availability Statement

The raw data supporting the conclusions of this article will be made available by the authors, without undue reservation.

## Ethics Statement

The studies involving human participants were reviewed and approved by Ethics Committee of the Shengjing Hospital of China Medical University. The ethics committee waived the requirement of written informed consent for participation.

## Author Contributions

XC and SL were involved in study design and data interpretation. SL and JZ involved in the data collection and data analysis. SL, JZ, and XL were involved in drafting the manuscript. SL and XL prepared figures and tables. All authors critically revised the manuscript and approved the final version.

## Funding

This study was supported by the Joint Plan of Key Research and Development Program of Liaoning Province (Grant Nos. 2020JH 2/10300137 and 2020JH 2/10300148) and the 345 Talent Project of Shengjing Hospital (Grant No. M0716).

## Conflict of Interest

The authors declare that the research was conducted in the absence of any commercial or financial relationships that could be construed as a potential conflict of interest.

## Publisher's Note

All claims expressed in this article are solely those of the authors and do not necessarily represent those of their affiliated organizations, or those of the publisher, the editors and the reviewers. Any product that may be evaluated in this article, or claim that may be made by its manufacturer, is not guaranteed or endorsed by the publisher.
