# Supplementary material for: Reduced Preoperative Glomerular Filtration Rate Is Associated With Adverse Postoperative Oncological Prognosis in Patients Undergoing Radical Nephroureterectomy for Upper Tract Urothelial Carcinoma: A Retrospective Cohort Study
Source: Front Surg. 2022 Apr 25;9:872273. doi: 10.3389/fsurg.2022.872273 (PMC9082599; doi:10.3389/fsurg.2022.872273)
Supplement: Supplementary file 1 [file Table_1.docx]

**Table 1** Subgroup analysis using potential confounders as the stratification variables.

| **Confounding factor category** | **N** | **eGFR group** | | | ***P* for trend** | ***P* for interaction** |
| --- | --- | --- | --- | --- | --- | --- |
|  |  | **eGFR≤45** | **45<eGFR≤60** | **eGFR＞60** |  |  |
| Age |  |  |  |  |  | 0.533 |
| <67 | 126 (47.9) | 1 (reference) | 0.22 (0.08~0.58) | 0.08 (0.03~0.19) | <0.001* |  |
| ≥67 | 137 (52.1) | 1 (reference) | 0.38 (0.16~0.92) | 0.18 (0.09~0.36) | <0.001* |  |
| Sex |  |  |  |  |  | 0.379 |
| Male | 136 (51.7) | 1 (reference) | 0.48 (0.15~1.49) | 0.24 (0.09~0.62) | 0.003* |  |
| Female | 127 (48.3) | 1 (reference) | 0.30 (0.14~0.65) | 0.10 (0.05~0.22) | < 0.001* |  |
| BMI |  |  |  |  |  | 0.613 |
| <23.95 | 130 (48.9) | 1 (reference) | 0.30 (0.11~0.81) | 0.10 (0.04~0.24) | <0.001* |  |
| ≥23.95 | 133 (51.1) | 1 (reference) | 0.37 (0.17~0.83) | 0.15 (0.08~0.34) | < 0.001* |  |
| Hypertension |  |  |  |  |  | 0.397 |
| No | 171 (65.0) | 1 (reference) | 0.44 (0.21~0.90) | 0.14 (0.07~0.27) | < 0.001* |  |
| Yes | 92 (35.0) | 1 (reference) | 0.16 (0.05~0.55) | 0.10 (0.04~0.26) | < 0.001* |  |
| Diabetes |  |  |  |  |  | 0.708 |
| No | 231 (87.8) | 1 (reference) | 0.36 (0.19~0.68) | 0.13 (0.07~0.23) | < 0.001* |  |
| Yes | 32 (12.2) | 1 (reference) | 0.18 (0.02~1.99) | 0.15 (0.03~0.78) | 0.052 |  |
| CHD |  |  |  |  |  | 0.133 |
| No | 218 (82.9) | 1 (reference) | 0.21 (0.09~0.48) | 0.11 (0.06~0.22) | < 0.001* |  |
| Yes | 45 (17.1) | 1 (reference) | 0.82 (0.28~2.36) | 0.24 (0.07~0.8) | 0.013* |  |
| Smoking history |  |  |  |  |  | 0.461 |
| No | 180 (68.4) | 1 (reference) | 0.27 (0.12~0.62) | 0.11 (0.05~0.23) | < 0.001* |  |
| Yes | 83 (31.6) | 1 (reference) | 0.56 (0.21~1.46) | 0.18 (0.08~0.4) | < 0.001* |  |
| History of BC |  |  |  |  |  | 0.277 |
| No | 256 (97.3) | 1 (reference) | 0.32 (0.17~0.6) | 0.13 (0.07~0.23) | <0.001* |  |
| Yes | 7 (2.7) | 1 (reference) | 4170530005.48 (0~Inf) | 1 (0~Inf) | 0.662 |  |
| Concomitant of BC |  |  |  |  |  | 0.789 |
| No | 249 (94.7) | 1 (reference) | 0.33 (0.17~0.65) | 0.14 (0.08~0.25) | < 0.001* |  |
| Yes | 14 (5.3) | 1 (reference) | 0.14 (0.01~2.06) | 0.04 (0~0.68) | 0.031* |  |
| Tumor laterality |  |  |  |  |  | 0.673 |
| Left | 144 (54.8) | 1 (reference) | 0.27 (0.11~0.64) | 0.13 (0.06~0.26) | < 0.001* |  |
| Right | 119 (45.2) | 1 (reference) | 0.45 (0.17~1.18) | 0.14 (0.06~0.36) | <0.001* |  |
| Tumor location |  |  |  |  |  | 0.486 |
| Renal pelvis | 105 (39.9) | 1 (reference) | 0.35 (0.1~1.24) | 0.24 (0.09~0.62) | 0.006* |  |
| Ureter | 140 (53.2) | 1 (reference) | 0.32 (0.14~0.71) | 0.09 (0.04~0.20) | <0.001* |  |
| Multiple | 18 (6.8) | 1 (reference) | 0.27 (0.03~2.24) | 0.08 (0.01~0.94) | 0.045* |  |
| Tumor size |  |  |  |  |  | 0.314 |
| <2.6cm | 130 (48.9) | 1 (reference) | 0.4 (0.17~0.91) | 0.10 (0.04~0.23) | <0.001* |  |
| ≥2.6cm | 133 (51.1) | 1 (reference) | 0.29 (0.11~0.76) | 0.17 (0.08~0.37) | <0.001* |  |
| Tumor grade |  |  |  |  |  | 0.540 |
| Low | 55 (20.9) | 1 (reference) | 0.43 (0.05~3.71) | 0.07 (0.01~0.56) | 0.006* |  |
| High | 208 (79.1) | 1 (reference) | 0.37 (0.19~0.72) | 0.16 (0.09~0.29) | <0.001* |  |
| pT stage |  |  |  |  |  | 0.580 |
| Ta,Tis,T1 | 93 (35.4) | 1 (reference) | 0.47 (0.17~1.35) | 0.14 (0.05~0.39) | <0.001* |  |
| T2 | 129 (49.0) | 1 (reference) | 0.32 (0.12~0.85) | 0.11 (0.05~0.24) | < 0.001* |  |
| T3 | 41 (15.6) | 1 (reference) | 0.23 (0.05~1.05) | 0.24 (0.07~0.86) | 0.069 |  |
| Lymph node status |  |  |  |  |  | 0.108 |
| Negative | 246 (93.5) | 1 (reference) | 0.42 (0.22~0.82) | 0.14 (0.08~0.27) | < 0.001* |  |
| Positive | 17 (6.5) | 1 (reference) | 0 (0~Inf) | 0.1 (0.01~0.87) | 0.023* |  |
| Surgical approach |  |  |  |  |  | 0.511 |
| Laparoscopic | 96 (36.5) | 1 (reference) | 0.4 (0.14~1.09) | 0.21 (0.08~0.55) | 0.002* |  |
| Open | 167 (63.5) | 1 (reference) | 0.34 (0.15~0.75) | 0.10 (0.05~0.21) | <0.001* |  |
| Chemotherapy |  |  |  |  |  | 0.189 |
| No | 176 (66.9) | 1 (reference) | 0.4 (0.18~0.89) | 0.19 (0.09~0.38) | <0.001* |  |
| Yes | 87 (33.1) | 1 (reference) | 0.29 (0.1~0.83) | 0.07 (0.03~0.18) | <0.001* |  |
| Hypoalbuminemia |  |  |  |  |  | 0.666 |
| No | 224 (85.2) | 1 (reference) | 0.35 (0.18~0.68) | 0.12 (0.07~0.23) | < 0.001* |  |
| Yes | 39 (14.8) | 1 (reference) | 0.25 (0.03~2.28) | 0.23 (0.06~0.87) | 0.041* |  |
| Anemia |  |  |  |  |  | 0.861 |
| No | 203 (77.2) | 1 (reference) | 0.38 (0.18~0.8) | 0.15 (0.07~0.29) | < 0.001* |  |
| Yes | 60 (22.8) | 1 (reference) | 0.28 (0.08~1.03) | 0.13 (0.04~0.37) | < 0.001* |  |

**P* < 0.05. eGFR: estimated glomerular filtration rate; BMI, body mass index; CHD; coronary heart disease; UTUC, upper tract urothelial carcinoma.
